# A Digital Twin Decision Support System for the Urban Facility Management Process †

**DOI:** 10.3390/s21248460

**Published:** 2021-12-18

**Authors:** Armir Bujari, Alessandro Calvio, Luca Foschini, Andrea Sabbioni, Antonio Corradi

**Affiliations:** Department of Computer Science and Engineering (DISI), University of Bologna, 40136 Bologna, Italy; alessandro.calvio2@unibo.it (A.C.); luca.foschini@unibo.it (L.F.); andrea.sabbioni5@unibo.it (A.S.); antonio.corradi@unibo.it (A.C.)

**Keywords:** Digital Twin, big data, geographic information system, smart city, Urban Facility Management, Apache Spark

## Abstract

The ever increasing pace of IoT deployment is opening the door to concrete implementations of smart city applications, enabling the large-scale sensing and modeling of (near-)real-time digital replicas of physical processes and environments. This digital replica could serve as the basis of a decision support system, providing insights into possible optimizations of resources in a smart city scenario. In this article, we discuss an extension of a prior work, presenting a detailed proof-of-concept implementation of a Digital Twin solution for the Urban Facility Management (UFM) process. The Interactive Planning Platform for City District Adaptive Maintenance Operations (IPPODAMO) is a distributed geographical system, fed with and ingesting heterogeneous data sources originating from different urban data providers. The data are subject to continuous refinements and algorithmic processes, used to quantify and build synthetic indexes measuring the activity level inside an area of interest. IPPODAMO takes into account potential interference from other stakeholders in the urban environment, enabling the informed scheduling of operations, aimed at minimizing interference and the costs of operations.

## 1. Introduction

Digital transformation is an essential element for urban governance, enabling the efficient coordination and cooperation of multiple stakeholders involved in the urban environment. In the last decade, the concept of the smart city has gained tremendous importance and public/private institutions have started thinking about innovative ways to develop solutions tackling the complexity of the various phenomena [1]. At their core, these solutions rely on IoT and ICT to realize the perception, control and intelligent services aimed to optimize resource usage, bettering the services for the citizens [1,2]. To this aim, real-time access to accurate and open data is central to unlocking the economic value of the smart city potential, opening a rich ecosystem to suppliers for the development of new applications and services [3].

Digital Twin (DT) technology has revived the interest in the smart city concept, understood as a digital replica of an artefact, process or service, sufficient to be the basis for decision making [4]. This digital replica and the physical counterpart are often connected by streams of data, feeding and continuously updating the digital model, used as a descriptive or predictive tool for planning and operational purposes. While the concept of Digital Twin is by no means new, recent advances in 5G connectivity, AI, the democratization of sensing technology, etc., provide a solid technological basis and a new framework for investment. As an example, ABI Research predicts that the cost benefits deriving from the adoption of urban DTs alone could be worth USD 280 billion by 2030 [5].

Recognizing these benefits, different public and private institutions have already started investing in the technology, and this fact is attested by several urban DT initiatives, aimed at addressing different and complex problems in the smart city context. In the H2020 DUET project, the Flanders region aims at building a multi-purpose DT platform addressing the impact of mobility on the environment, to reduce the impact on human health [6]. In the same project, the city of Pilsen is building a proof-of-concept DT focusing on the interrelation between transport and noise pollution. The pilot aims to demonstrate the concept of the technology across transport and mobility, urban planning and the environment and well-being limits [7].

Recently, Bentley Systems and Microsoft announced a strategic partnership to advance DT for city planning and citizen engagement [8]. This partnership materialized in 2020 in a collaboration with the city of Dublin, pursuing the development of a large-scale urban DT used as a support tool for citizens to engage, from the safety of their own homes, in new development projects in their local communities.

It is evident that the success of an urban Digital Twin is directly impacted by the quality and quantity of the data sources that it relies on to model the physical counterpart. The modeling component, being either visual, e.g., based on a dashboard showing a consolidated view of the data, or data-driven, e.g., AI or statistical, is another core element of the framework. In this work, we discuss a concrete DT solution for the Urban Facility Management (UFM) process. The process under scrutiny comprises multiple stakeholders acting on a shared environment, e.g., different companies involved in the maintenance of various urban assets, each having a local view of the overall maintenance process. Their view on the activities is periodically consolidated in a joint conference called by the municipality, where mid-to-long-term operational details are discussed and a global, coarse-grained view of the process is established.

The scope and aim of the project is to showcase the use of DT technology as a decision support system, guiding UFM operators in their activity through the use of a rich set of correlation tools depicting the activities inside an area of interest. The system is equipped with a scheduling functionality, consulted to find feasible schedules for maintenance interventions, while minimizing some predetermined indexes, e.g., disturbance on mobility, interferences with other planned city events, etc. From a technological viewpoint, the Interactive Planning Platform for City District Adaptive Maintenance Operations (IPPODAMO [9]) is a proof-of-concept DT consisting of a (distributed) multi-layer geographical system, fed with heterogeneous data sources originating from different urban data providers. The data are initially staged at ingestion points, dedicated ingress machines, where they undergo syntactic and semantic transformations, and are successively forwarded to a big data processing framework for further refinements. The data are subject to different algorithmic processes, aimed at building a coherent view of the dynamics inside an area of interest, exploited by the UFM operated to make informed decisions on potential future maintenance operations. This work builds on a prior work [10], extending the study with a detailed discussion of some core system components, showcasing the advanced capabilities of the decision support system.

The article is organized as follows. Section 2 provides a concise background on the concepts and technological ecosystem adopted in this work. Section 3 briefly describes some data sources considered in the project, followed by a high-level functional overview of the multi-layer geographical system. Section 4 presents two distinct big data processing pipelines, providing some insights into their implementation and performance trends. While preserving the general aspect of our study and without loss of generality, in Section 5, we present the use cases and functionalities currently targeted by the platform. Finally, Section 6 draws the conclusions, delineating some future work.

## 2. Background

This section provides a brief overview of the Digital Twin concept, presenting its main building blocks and relating them to IPPODAMO. Next, we provide a concise and contextual survey of big data computing frameworks, part of the technological ecosystem adopted in this work.

### 2.1. Digital Twin Concept

Grieves is recognized to be the first that coined the term Digital Twin, using it to describe the digital product lifecycle management process [11]. Since then, thanks to the availability of modern computing platforms, engineering and management paradigms and practices, the term has gained in popularity, promoting DT technology as a key enabler of advanced digitization.

To date, many such applications have been successfully implemented in different domains, ranging from manufacturing [12] and logistics [13] to smart cities [14], etc. However, currently, there is no common understanding of the term, and in this respect, a taxonomy would help to demarcate the conceptual framework.

To this end, the authors in [4] provide a classification of the individual application areas of Digital Twins. As part of a comprehensive literature review, they develop a taxonomy for classifying the domains of application, stressing the importance and prior lack of consideration of this technology in information systems research. Building on this, the authors of [15] undertake a structured literature review and propose an empirical, multi-dimensional taxonomy of Digital Twin solutions, focusing on the technical and functional characteristics of the technological solution. As an add-on, the proposal allows for the classification of existing Industry 4.0 standards enabling a particular characteristic.

What unites the various DT approaches is the presence of some key technological building blocks that need to be considered and are crucial to its success.

1. Data link: the twin needs data collected from its real-world counterpart over its full lifecycle. The type and granularity of the data depend on the scope and context of deployment of the technology.
  IPPODAMO relies on a multitude of heterogeneous data sources, available at different granularities. Each data source has a dedicated (automatic) ingestion process used to transform the data, enriching the IPPODAMO data layer. For more information on the data sources and some computational aspects, we refer to the reader to Section 3 and Section 4, respectively.
2. Deployment: the twin can be deployed and span the entire cloud-to-thing continuum, starting from the thing (IoT devices), the edge and/or the cloud. The specific deployment criteria depend on the scenario requirements, typically based on latency/bandwidth and/or security/privacy constraints.
  IPPODAMO is not directly involved in the raw data collection process. The system relies on third-party data providers, which collect, extract and fetch the data in dedicated ingress, cloud-backed machines. The raw data are anonymized and temporally retained in the system.
3. Modeling: the twin may contain different and heterogeneous computational and representational models pertaining to its real-world counterpart. This may range from first-principle models adhering to physical laws; data-driven; geometrical and material, such as Computer-Aided Design/Engineering (CAD, CAE); or visualization-oriented ones, such as mixed-reality.
  Our Digital Twin solution provides a consolidated view of heterogeneous urban data (descriptive Digital Twin), while at the same time, relying on historical and (near-)real-time data to perform near-to-mid-term predictions on the activity indexes (predictive Digital Twin). This allows operators to perform simulation scenarios aimed at minimizing predetermined indexes during routine and scheduled interventions. More information on the use cases and, in particular, the UFM scheduling is provided in Section 5.
4. APIs: the twin needs to interact with other components, e.g., twins in a composite system or an operator consulting the system. To facilitate these interactions, various APIs must be available to allow for information collection and control between the twin and its real-world counterpart.
  IPPODAMO presents the UFM operator an intuitive, high level user interface abstracting low-level system interfaces. At the same time, IPPODAMO has a programmatic interface, exposing well-defined ReST APIs through which other systems and operators could integrate and interact. The rationale behind this choice is to enable future potential integrations of IPPODAMO into a larger, federated ecosystem of smart city platforms. This aspect of the study is beyond the scope of this article.
5. Security/privacy: considering the role and scope of the DT, physical-to-digital interactions require security/privacy mechanisms aimed at securing the contents of the twin and the interaction flows between the twin and its physical counterpart.
  The solution is deployed on a state-of-the-art virtualized environment equipped with all the necessary security features. To this end, different administrative levels are provisioned, for accessing both the virtualized system and the system functionalities.

In the following, we provide a concise survey on some big data computing frameworks, motivating the choice of the identified technological ecosystem.

### 2.2. Big Data Computing

Smart cities generate and require, more than often, the collection of massive amounts of geo-referenced data from heterogeneous sources. Depending on the process and purpose of the system(s), these data must be quickly processed and analyzed to benefit from the information. To this end, big data computing frameworks have gained tremendous importance, enabling complex and online processing of information, allowing us to gain insights about phenomena in (near-)real time.

Cluster computing frameworks such as Apache Hadoop, based on the MapReduce compute model, are optimized for offline data analysis and are not ideal candidates for fast and online data processing due to the overhead of storing/fetching data at intermediate computation steps [16]. Current efforts in the relevant state-of-the-art have shifted toward promoting a new computing paradigm referred to as Stream Processing [17]. This paradigm adheres to the dataflow programming model, where computation is split and modeled as a directed acyclic graph and data flow through the graph, subjected to various operations [18]. Micro-batching represents a middle ground in this continuum: the idea is to discretize the continuous flow of data as a set of continuous sequences of small chunks of data, delivered to the system for processing. In this context, frameworks such as Apache Spark adopt this philosophy [19]. While Apache Spark is not a purely streaming approach, it introduces a powerful abstraction—the resilient distributed dataset (RDD [20])—allowing for distributed and in-memory computation. This programming abstraction supports efficient batch, iterative and online micro-batching processing of data, and it is a perfect match for the dynamics of our UFM scenario. Moreover, the cluster computing platform offers a wide range of libraries and computation models, as well as the geographical extensions needed to handle spatial data [21].

## 3. The IPPODAMO Platform

In this section, we start by presenting the data sources used by the Digital Twin, discussing their spatial and temporal characteristics. Next, we provide a high-level overview of the functional components comprising the technical solution.

### 3.1. Data Sources

IPPODAMO relies on a multitude of heterogeneous data sources provided, in part, by the project partners, including a telco operator and a company operating in the UFM sector.

**Figure 1:**
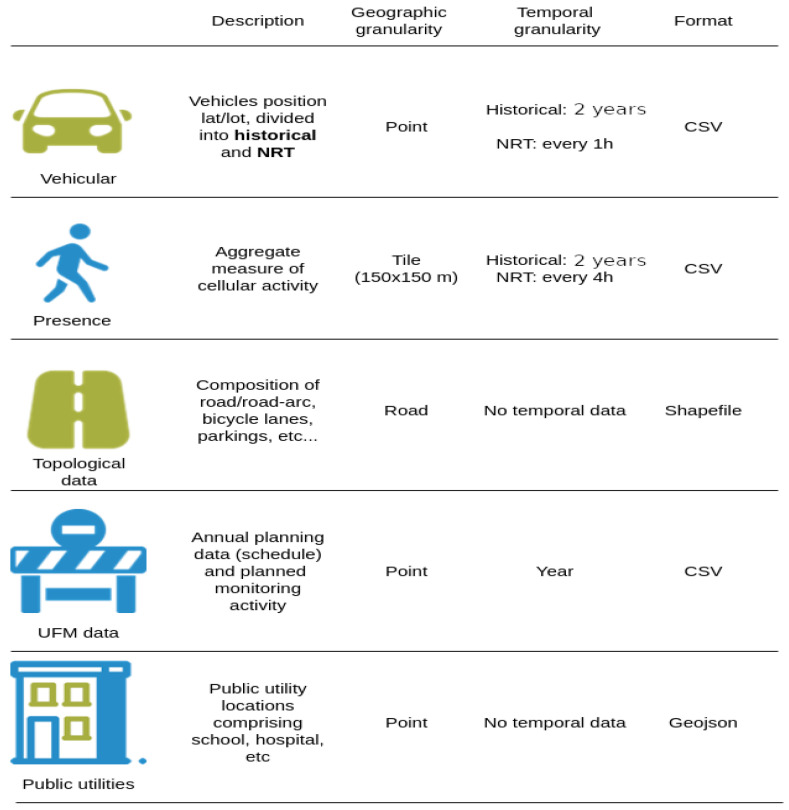
IPPODAMO data sources.

Referring to Figure 1, the twin relies on (anonymized) vehicular and human presence data, combined to extract a measure of the activity inside an area of interest. The data sources are geo-referenced, embodying different levels of granularity, subject to different data processing steps and aggregation procedures aimed at building a composable activity level index (refer to Section 5). At the time of writing, the system has processed and stores two years of historical data and actively processes (near) real-time updates from each data source. These data have important value and enable IPPODAMO to gain (near-)real-time insights into the activities, but also to simulate near-to-mid-term evolutions of the activity level by exploiting the historical data.

Other important data relate to the UFM process itself, which comprises data generated from: (i) the urban monitoring activity, e.g., the status of an urban asset assessed periodically through field inspections, (ii) annual planning and scheduled operations, e.g., repair interventions, (iii) geographical data concerning public utilities such as hospitals, schools, cycling lanes, etc. The data are provided and updated by the company operating in the UFM sector, having a vested interest in accurately depicting and monitoring the status of the urban assets.

Additional data sources are available and extracted from the open data portal, curated and maintained by the municipality of Bologna, Italy, and these include: (i) city events and (ii) other public utility maintenance operations.

All the above-mentioned data sources undergo dedicated processing steps and are stored in a logically centralized system, providing a consolidated and multi-source data layer. A rich set of visualizations can be built, guiding the UFM operator in their work. At the same time, more advanced functionalities, relying on the historical data to predict future evolutions of the phenomena inside an area of interest, are possible, and this topic is discussed in Section 5.

### 3.2. Technological Ecosystem

**Figure 2:**
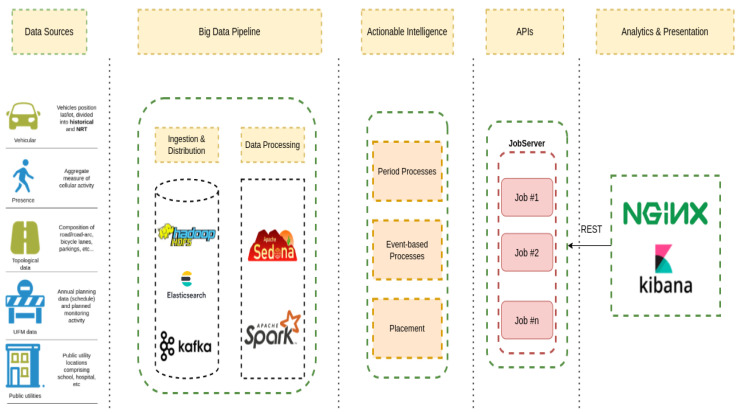
Technological components of the IPPODAMO platform.

The platform (Figure 2) is structured in four main conceptual layers: (i) the ingestion layer, which interacts with the data providers, continuously acquiring new data, performing syntactic transformations, pushing them upwards for further refinements; (ii) the big data processing layer, which performs semantic transformation and enrichment of raw data fed from the ingestion points; (iii) the storage layer, providing advanced memorization and query capability over (near-)real-time and historical data, and (iv) the analytics layer, presenting to the customer an advanced (near-)real-time layer with query capabilities, aggregate metrics and advanced representations of data.

For each data source used by the Digital Twin and, in particular, for the vehicular and presence data, there is a custom ingestion process tasked with reading the raw data, performing some syntactic transformations and pushing them towards the big data cluster. The various data sources are retrieved and pushed in parallel to specific Kafka topics, which identify also the semantic processing pipeline. Indeed, to enable the reliable and fast delivery of data from the ingestion points to our analytics and storage platform, we rely on Apache Kafka, an open-source, distributed, message-oriented middleware [22]. This choice is driven by the capabilities of this platform, including, but not limited to, its capability to gracefully scale the processing in the presence of high-throughput and low-latency ingress data. This matches the requirements of our domain, where data are constantly and periodically collected from many heterogeneous sources, e.g., vehicle black boxes, cellular, public transport, etc.

To provide advanced and fast processing capabilities for spatial data, the technological stack integrates and relies on Apache Sedona [23]. Apache Sedona is a distributed processing library, which builds on the in-memory computation abstraction of the Apache Spark framework and is able to provide advanced and fast queries over spatial data. Thanks to this spatial processing framework, we are able to blend and elaborate different data sources, creating rich representations of various phenomena in an area of interest.

Once the data have been processed, they are stored in Elasticsearch, a fast and scalable no-SQL database with advanced capabilities of indexing and querying key-value data [24]. Thanks to the advanced integration between Spark and Elasticsearch, we can use the solution as both a data provider and as a distributed storage system. The data are subject to further refinements and algorithmic processes aimed at creating different layers of aggregations, calculating synthetic indexes, etc., which are then used by the planner functionality to identify suitable time intervals during which to schedule urban operations. The raw data and information extracted from the data are presented to the end-users in different forms through advanced visualization dashboards available through Kibana, part of the Elasticsearch ecosystem. Last is the JobServer component, an optimized RESTful interface for submitting and managing Apache Spark tasks [25]. A job request, e.g., for a suggestion on a maintenance operation schedule, is a JSON-formatted request, triggering the execution of a specific algorithm resulting in a JSON-formatted output visualized through a web-based user interface. This component composes part of the IPPODAMO programmatic API, which could be used to integrate the solution into a larger, federated ecosystem of platforms in a smart city context.

## 4. Big Data Processing

In this section, we provide some technical details on the big data processing pipelines dedicated to the transformation and enrichment of incoming raw data. Next, we present and analyze the performance trend of two distinct data processing pipelines, providing in-depth insights into some system mechanisms.

### 4.1. Processing Pipeline(s)

The data sources introduced in Section 3 are subject to different processing pipelines due to the inherent syntactic and semantic differences that they embody. Concerning the vehicular and presence data, of relevance to the UFM process is the measure of the activity level inside a particular area in time. To this end, both pipelines are finalized to implement a counting technique measuring the activity volume. The geographical granularity of the presence data is accounted on a tile basis, a square-shaped geographical covering an area of 150 m × 150 m, while the vehicular data are point data—latitude and longitude—and can be accounted for at any meaningful granularity. These data resolutions are imposed by the data provider. It is important to note that in scenarios where both vehicular and presence data need to be accounted for, the granularity of this aggregation operation can be a tile or a multiple of tile entities.

**Figure 3:**
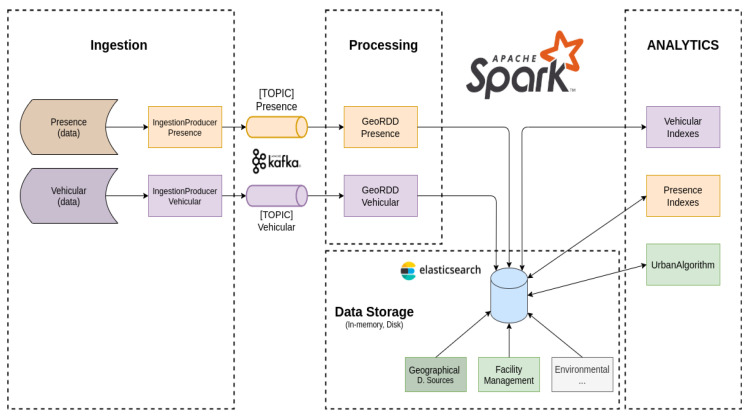
IPPODAMO (big) data processing pipelines and information flow.

Referring to Figure 3, both vehicular and presence data sources are initially subjected to some syntactic transformations before being forwarded from the Kafka producer, simplifying the ingestion process on the Spark cluster. From here on, the data are subject to semantic transformation processes, enriching the source data with relevant geographical information. In particular, the last operation in the pipeline accounts for the activity index information, retaining it in a distributed memory support. This operation should be done in a fast and efficient way; otherwise, it risks becoming a bottleneck for the overall system, delaying the ingestion performance.

To this end, we rely on a spatial partitioning mechanism, dividing the interest area among the cluster nodes. The area of interest to the project is initially saved in the underlying distributed file system and programmatically loaded and partitioned using the GeoHash spatial partitioning scheme [26]. This allows us to distribute topological data and index thereof among the cluster nodes. Indeed, in scenarios where ingress data are uniformly distributed inside the area of interest, this allows us, on average, to equally distribute and scale the computation among available cluster nodes. In particular, the data are accounted for by performing distributed point-in-polygon (PiP) and k-Nearest-Neighbor (kNN) operations, and these operations are enclosed by the *GeoRDDPresence* and *GeoRDDVehicular* functional components, respectively, for the presence and vehicular data. The data, once retained, are then subjected to additional processes, aggregating and slicing the data in the time and space domains (refer to Section 5). Other sources of information are those containing topological information concerning urban assets such as cycling and bus lanes, hospitals, schools, etc., and open data offered by the municipality, e.g., city events. Topological data have a dedicated batch processing pipeline; at its crux is a geo-join operation, aimed at enriching the IPPODAMO baseline topological map with additional information on urban assets. A dedicated update procedure is available whereby old information is discarded and only the fresh information is considered.

Last are the data sources containing operational information on the facility management process. UFM data have spatial and time information and are currently handled by a similar processing pipeline to the topological data.

### 4.2. Performance Analysis

The periodicity of the individual data sources imposes some operational constraints on the data processing pipelines, and this consideration applies to the vehicular and presence data. As an example, when a new batch of vehicular data enters the system, the corresponding end-to-end processing pipeline comprising the various syntactic and semantic transformations should take no more than 60 min (refer to Figure 1). Failure to do so would create a backlog of data that increases over time, slowing down the ingestion performance.

In particular, among the operations shown in Figure 3, the one embodying the highest computational burden is the *GeoRDDVehicular* operation, which requires the execution of a kNN operation for each data point present in the hourly dataset. We report that the end-to-end sequential processing of an hourly vehicular dataset containing, on average, 15,000 records (trips) often results in a violation of its operational constraint.

**Figure 4:**
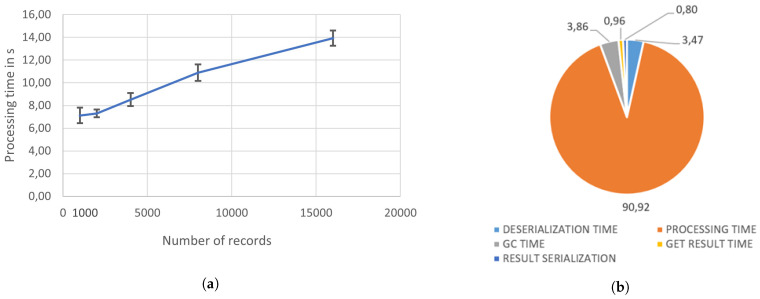
Vehicular processing pipeline performance. The experiments were carried out in a testbed comprising 4 VMs—1 driver and 3 workers—each equipped with 8 vCores, 32 GB vRAM and 150 GB data SSD support. (**a**) Overall processing time under varying number of ingress records. (**b**) Processing time decomposition for the 15,000 record configuration.

To address the issue, we leverage some specific constructs of the Apache Sedona framework, aimed at distributing the computational effort among the various cluster nodes. As anticipated, the proposed solution makes use of the (i) GeoHash spatial partitioning scheme, allowing the partitioning of a geographical area of interest among cluster nodes, and the (ii) broadcast primitive implementing a distributed, read-only shared memory support. In particular, the ingress vehicular dataset is enclosed as a broadcast variable, shared among worker nodes, where each node is responsible for the computation of a kNN operation over a subset of the points contained in the original dataset. This approach allows the parallel computation of individual kNN operations, whose outcome is later merged and retained in ElasticSearch. Figure 4 shows the performance trend of the optimized vehicular data processing pipeline. In particular, Figure 4a plots the processing time with a varying number of input records. The resulting trend is monotonic, allowing for the timely processing of the input dataset, adhering to the operational constraints imposed by the data source periodicity. Figure 4b puts the processing time into a greater context, accounting for additional operations occurring before and after the *GeoRDDVehicular* processing step, comprising data (de)serialization and output communication to the driver node, with the kNN processing step accounting for nearly 91% of the processing time.

## 5. A Decision Support System

At its core, the IPPODAMO platform serves as a decision support system, aiding UFM operators in their daily activity. In this section, we start by discussing a set of identified use cases best targeting the needs of the UFM operator. Next, we discuss the concept behind the activity level index, a synthetic index used by some underlying system functionalities. Then, we provide a brief description of the algorithmic details of the scheduler functionality, along with a validation study showcasing its capabilities.

### 5.1. Use Cases

While preserving the general aspect of our study and without loss of generality, we identified two broad use cases, which showcase the capabilities of the solution in the following directions:

- UC#1: Rich (comparative) analysis by providing a set of configurable visualizations, used to quantify and visualize the activities inside an area of interest.
- UC#2: UFM scheduling functionality guiding the placement of maintenance operations in time.
- UC#3: Quantitative evaluation of the annual planning interventions.

Concerning the first use case, the system provides a rich set of configurable visualizations, allowing the UFM operator to consult and confront the historical and current trend of data inside an area of interest. The second use case aims to provide the UFM planner with a proactive decision tool, guinding the scheduling decisions. The last use case allows the platform administrator to perform an *a posteriori* evaluation of the annual planning schedule, dictated by the data that IPPODAMO has ingested. Through this functionality, we would like also to be able to perform a qualitative evaluation of the algorithmic decisions made by IPPODAMO, confronting them with the knowledge of the UFM specialist. At the core of these use cases is the activity level index, which is discussed in the following section.

### 5.2. Activity Level Index

Once the data are processed, they are stored in ElasticSearch and are subjected to further periodic and event-based algorithmic processes, aggregating and slicing the data in the time and space domains. Intuitively, the activity index is a measure of the activity level inside an area of interest.

At first, vehicular data are stored at their finest granularity, contributing to the traffic volume in a specific point of the underlying road topology. These point-wise data are aggregated and accounted on a road-arch basis, a constituent of the road topology. The data are also sliced in the time domain, accounting for the daily traffic volume and the traffic volume on some configurable rush hours. Once aggregated, the data are normalized and stored at different scales, e.g., for better visualization. This computed quantity constitutes the activity level index derived from the vehicular data.

The human presence data are subjected to a similar workflow. The difference is in the granularity (tile entity) that the data are accounted for. Recall that this granularity is imposed on us by the data provider. Once the individual indexes are computed, they can be composed and weighted according to some criteria. The current implementation allows an operator to simulate different scenarios by specifying the weights accordingly.

These indexes are periodically updated and maintained, and they constitute the input for the, e.g., UFM scheduler. Currently, three implementations for the activity level index are available, and the planners’ behavior is parametric on the index type:

1. Raw index: scaled activity level computed from the raw data. This index has only look-back capabilities and is used by the UFM specialist to compare the goodness of the performed annual activities. This index is connected to the functionality of UC#3, via which we would like to obtain a qualitative evaluation of the platforms’ operations.
2. Smoothed index: a window-based, weighted average index adopted to filter noise and potential erratic behavior of the raw index. Similarly, this index has only look-back capabilities, serving as a starting point for more sophisticated types of indexes.
3. Predictive index: this index has look-ahead capabilities, leveraging the past to predict near-to-mid-term, e.g., monthly-based evolution, activity levels in a specified geographical area. This index is used to create future hypotheses for scheduling operations.

Concerning the predictive index, we rely on state-of-the art algorithms, capable of inferring seasonality and local phenomena from the data. Currently, we are evaluating some practical design considerations that can occur in a dynamic and time-varying scenario such as ours.

### 5.3. UFM Scheduling Algorithm

This functionality is used in the first use case, and aids the UFM operator in searching for a suitable timeframe during which schedule a maintenance intervention. A maintenance operation may consist of a minor/major repair operation of an urban asset; it has fixed coordinates in space, a predicted duration, and an optional timeframe in which it needs to be scheduled. The scheduling criteria vary, and, depending on the objective, one would like to avoid or minimize disturbance to nearby activities. To this aim, the system has the capability to express and consider all these and other constraints when performing the search for a suitable timeframe.

Once a request is issued, the system receives all the constraints expressed by the operator, including the list of attributes, e.g., coordinate, expected duration, etc., for each intervention. The algorithm then exploits the geographical coordinates to compute the activity level index inside an area of interest and gathers all the potential interferences with nearby ongoing activities. To this aim, the functional component relies on the Spark SQL library and geographical primitives available in Apache Sedona. Recall that the algorithms’ behavior is parametric on the index type, and different types of indexes are available. The final outcome of this computation is the construction of the index history and its hypothetical evolution in time.

Once the activity level index is computed, and all interferences have been retrieved, a final index is processed for the time horizon under consideration served as input to the scheduling algorithm. The exact algorithmic details and final index composition are beyond the scope of this article.

### 5.4. Discussion

**Figure 5:**
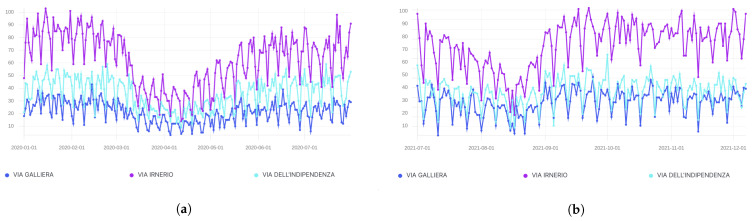
Trend of the vehicular index—scale [0, 100] for better visualization—in the pre-COVID-19, during and post-COVID-19 period. (**a**) Timeline comprising the COVID-19 period. (**b**) Post-COVID-19 period.

Figure 5 shows a comparative view through which the UFM operator can assess the vehicular activity in configurable timeframes and areas. These views fall inside the functionalities provisioned in the second use case, and are all configurable via dedicated high-level graphical interactions. Once the configuration has been set up, a Kibana view is generated and mirrored in the frontend.

**Figure 6:**
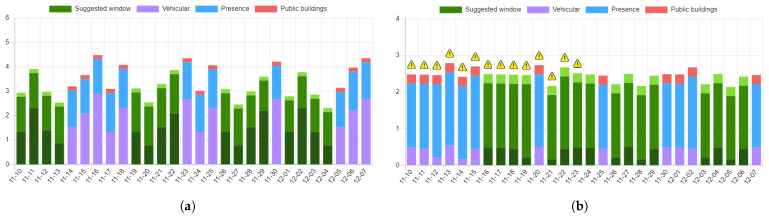

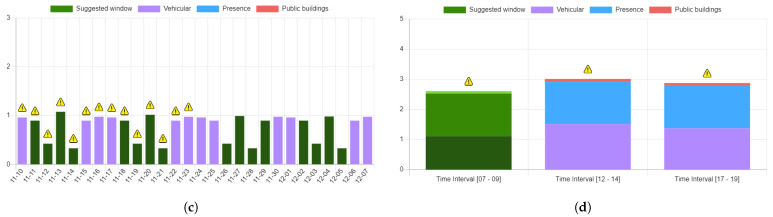
The UFM scheduler result, providing the operator with potential timeframes (green color) during which to schedule a specific maintenance operation. The final activity index is decomposed in all its constituent values, contributing to the final index. The analysis considers a period of one month, starting from 10 November 2021. (**a**) Equal cost timeframes proposed by the scheduler for a maintenance operation in Via Indipendenza X, Bologna, Italy. (**b**) Identified non-binding interference for a maintenance operation in Via Zamboni, Bologna, Italy, in the interval [10, 23]. (**c**) Equal cost timeframes, vehicular data only, proposed by the scheduler for a maintenance operation in Via Zamboni. (**d**) Scheduling of an urgent intervention in Via Zamboni, relying on the next-day prediction of the activity index.

Figure 6 shows some sample outputs generated by the UFM scheduler functionality provisioned in the second use case. For this assessment, the algorithm relies on the predictive index and co-locality information identifying nearby interferences, e.g., city events, other public utility maintenance operations announced by the municipality, etc. A maintenance operation has a specific location in space, and a duration in time that could span several days or hours (Figure 6). Indeed, as already discussed, the data are sliced in the time domain: in Figure 6a, the algorithm exploits the daily predictive index to position a maintenance operation in time, while, in Figure 6d, the maintenance operation is, generally, positioned in a series of consecutive (pre-configured) time intervals. The IPPODAMO interface allows an operator to specify this additional search criterion.

In all the charts, the lines denoted in green identify the minimum cost schedules along with potential identified interferences, gathered and reported via the user interface. It is noteworthy to point out that, in the current implementation, interferences do not contribute to the index, but rather serve as additional information guiding the UFM operator to make an informed decision (Figure 6b,c). In addition, the interface allows the UFM operator to customize the weights of the individual parameters, e.g., as shown in Figure 6c, where the only quantity contributing to the index is that derived from the vehicular data.

## 6. Conclusions

In this work, we presented a Digital Twin solution for the Urban Facility Management process in a smart city context. IPPODAMO is a multi-layer, distributed system making use of a multitude of heterogeneous data sources to accurately depict and predict the dynamics inside a geographical area of interest. The decision support system consists of a wide variety of visualizations, including a scheduler functionality, aiding UFM operators in their maintenance placement activity.

Currently, the solution is being tested in a real operational scenario, and we are studying emergent software behavior, identifying near-to-mid-term directions to extend the software. Of paramount importance is the capability to quantify the benefits of the solution through measurable KPIs. To this end, we are collaborating with the private sector and structuring a qualitative data gathering process that could serve as a basis for the value proposition of the proposal. 

## Data Availability

Not applicable.
